# Physical Activity Is Associated With Decreased Epigenetic Aging: Findings From the Health and Retirement Study

**DOI:** 10.1002/jcsm.13873

**Published:** 2025-06-13

**Authors:** Farah Ammous, Mark D. Peterson, Colter Mitchell, Jessica D. Faul

**Affiliations:** ^1^ Survey Research Center, Institute for Social Research University of Michigan Ann Arbor Michigan USA; ^2^ Department of Physical Medicine and Rehabilitation University of Michigan Medicine Ann Arbor Michigan USA; ^3^ Population Studies Center, Institute for Social Research University of Michigan Ann Arbor Michigan USA

**Keywords:** DNA methylation, epigenetic aging, life‐course modelling, physical activity

## Abstract

**Background:**

Epigenetic aging measures or clocks are DNA methylation‐based indicators of biological aging, linked to health outcomes and disease risk. Physical activity and exercise may influence epigenetic aging, suggesting a pathway through which it promotes healthier aging and reduces chronic disease burden. In this study, we assessed the association between self‐reported moderate‐to‐vigorous physical activity and epigenetic age acceleration (EAA) in participants of the Health and Retirement Study, followed biennially for 12 years from 2004 to 2016.

**Methods:**

Leukocyte DNA methylation was measured from venous blood samples collected in 2016 and second‐generation epigenetic clocks (GrimAge, PhenoAge and DunedinPACE) were used to assess EAA. Physical activity was assessed at each wave, with participants reporting vigorous activity at least once per week or moderate activity more than once per week or more categorized as ‘physically active’. We used weighted linear regression models for cross‐sectional analysis in 2016. Additionally, we used a structured life‐course modelling approach (SLCMA) to assess how the timing of physical activity is associated with EAA, testing both wave‐specific physical activity measures and an accumulation measure indicative of sustained physical activity over the follow‐up period.

**Results:**

In 2016, 58% of the participants were classified as physically active. In cross‐sectional analysis, physically active participants had lower EAA than inactive participants: −1.26 (95% confidence interval (CI): −1.59, −0.93) years for GrimAge acceleration, −1.70 (95% CI: −2.26, −1.15) for PhenoAge acceleration and −0.05 (95% CI: −0.06, −0.04) years per chronological year for DunedinPACE, adjusted for age, gender, race/ethnicity, education, total wealth and current smoking status. The associations attenuated slightly but remained significant after further adjusting for body mass index, mobility limitations and chronic disease diagnosis. We found evidence of effect modification in the association between physical activity and EAA by social factors, with stronger associations in males compared to females for DunedinPACE (*P*_interaction_ = 0.04) and a positive association between physical activity and increased EAA among Hispanics compared to non‐Hispanic Whites for GrimAge (*P*_interaction_ = 0.009). Our longitudinal analysis using SLCMA identified both accumulation and most recent physical activity in 2016 as the strongest predictors of EAA.

**Conclusions:**

Our findings highlight physical activity as a robust factor associated with slower epigenetic aging, with both accumulation and concurrent physical activity as the strongest predictors. These results underscore the role of physical activity in promoting healthier biological aging, suggesting its potential as a target for interventions aimed at mitigating age‐related health decline.

## 1 Introduction

Epigenetic aging, characterized by age‐associated changes in DNA methylation (DNAm) is increasingly recognized as an important biomarker of biological aging and a predictor of morbidity and functional decline [1, 2, 3]. Physical activity influences the expression of exercise‐responsive genes and modulates inflammatory processes, which may contribute to its effects on epigenetic aging [4, 5, 6]. Observational and intervention studies have shown that physical activity is associated with slower epigenetic aging, supporting its potential as an intervention for promoting healthy aging [7, 8, 9, 10, 11]. These studies have largely relied on cross‐sectional associations in cohort studies [7, 8, 9] or intervention studies of structured exercise with short follow‐up durations and smaller number of healthy participants [10, 11]. Longer term studies in diverse and larger samples remain needed to help understand how physical activity is associated with epigenetic aging.

Over the past decade, several epigenetic aging clocks have been developed. Among these, second‐ and third‐generation clocks, trained on physiological and clinical measures, better capture interindividual variability in underlying biological aging processes compared to first‐generation clocks, which were trained solely on chronological age [12]. Some of these clocks include PhenoAge, GrimAge and DunedinPACE (PACE). PhenoAge, trained on clinical biomarkers and chronological age, predicts mortality, physical function, cognitive performance and other age‐related outcomes [13]. GrimAge, developed using DNAm surrogates for plasma proteins and smoking pack‐years, is strongly linked to lifespan and age‐related conditions [14, 15, 16]. The PACE clock, the most recent of the three, was trained on longitudinal changes in biomarkers across multiple organ systems and is associated with morbidity, disability and mortality [17, 18].

In this study, we investigated the association between self‐reported physical activity, measured over a 12‐year period, and three epigenetic age acceleration (EAA) measures estimated using PhenoAge, GrimAge and PACE in a large and US representative cohort of participants from the Health and Retirement Study (HRS). In addition to cross‐sectional analysis, we employed a structured life‐course modelling approach (SLCMA) to formally and efficiently test how timing of activity is associated with epigenetic aging [19]. Specifically, we tested whether EAA is best explained by physical activity concurrently with epigenetic aging (i.e., in 2016), prior measures of physical activity (2004–2014), or by an accumulation measure indicative of sustained physical activity over the follow‐up period.

## 2 Methods

### 2.1 The HRS

The HRS is an ongoing national longitudinal panel study that has surveyed a sample of approximately 20 000 US adults over the age of 50 every 2 years since 1992 [20]. The HRS is sponsored by the National Institute on Aging and is conducted by the University of Michigan (NIA U01AG009740). The goal of the HRS is to continually represent the entire US population over the age of 50. Starting in 1998, the HRS has employed a steady state design, refreshing its sample with a new 6‐year birth cohort every 6 years. Follow‐up is completed via enhanced face‐to‐face interviews every 2 years for half of the sample or via phone interviews for the other half. This study included participants who provided blood samples as part of the Venous Blood Study in 2016 for DNAm assessment in leukocytes (*n* = 4018) [21]. Compared to the overall HRS sample, participants with DNAm assessment were older, had more chronic disease, and fewer were current smokers, but their physical activity levels, BMI and mobility limitations did not differ significantly (Table S1).

In this study, we limited our sample to participants with sampling weights for a sample that is representative of US adults aged 56 years and older with information on physical activity (*n* = 3873).

### 2.2 DNAm and EAA

Leukocyte DNAm measurement was done using the Infinium Methylation EPIC BeadChip v1.0. Details on DNAm measurement and quality control have been previously described [22]. Counts of white blood cell types (B cells, CD8 + T cells, naïve CD8 + T cells, natural killer cells and monocytes) were estimated using flow cytometry [23]. PhenoAge is estimated using 513 DNAm sites known as CpGs. It is trained on a composite clinical measure of phenotypic age, which is derived from chronological age and nine biomarkers, including albumin, creatinine and serum glucose [13]. GrimAge was developed by first establishing DNAm‐based surrogate measures for a set of plasma proteins and smoking pack‐years using 1030 CpG sites. These surrogate measures were then regressed on time‐to‐death, and their linear combination was then used to estimate GrimAge [14]. For both GrimAge and PhenoAge, EAA was defined as the residual from a regression of epigenetic age on chronological age. These residuals, GrimAA and PhenoAA, quantify the discrepancy between biological and chronological aging, where a one‐unit increase corresponds to a 1‐year increase in biological aging. PACE estimates the rate of biological aging and was developed using longitudinal data on physiological decline across multiple organ systems and trained to capture variation in aging trajectories [17]. For PACE, a one‐unit increase reflects a rate of biological aging per year of chronological age, with values greater than 1 indicating accelerated aging. Both GrimAge and PhenoAge are publicly released by the HRS [22]. The PACE clock was estimated using the PACEProjector provided by Belsky and colleagues [17].

### 2.3 Physical Activity

HRS participants were asked about the frequency of vigorous, moderate or mild physical activity in their daily lives at each follow‐up wave between 2004 and 2016 [24]. For vigorous activity, participants were asked ‘how often do you take part in sports or activities that are vigorous, such as running or jogging, swimming, cycling, aerobics or gym workout, tennis, or digging with a spade or shovel?’ For moderate activity, participants were asked ‘how often do you take part in sports or activities that are moderately energetic such as, gardening, cleaning the car, walking at a moderate pace, dancing, floor or stretching exercises’. Participants were also asked about mild activity, which included activities such as vacuuming, laundry and home repairs. For each level of physical activity, participants reported whether they took part in the activity *every day*, *more than once a week*, *once a week*, *1–3 per month* or *never*.

Current US guidelines recommend between 150 and 300 min of moderate‐intensity aerobic activity, 75 and 150 min of vigorous‐intensity aerobic activity per week or equivalent combination of moderate‐ and vigorous‐intensity activity, for substantial health benefits in adults [25]. The HRS survey does not collect information on exercise duration, limiting our ability to fully assess aerobic activity levels of adherence to guidelines. For this study, we derived a binary measure of physical activity, **PA‐2016**, that targets moderate‐ and vigorous‐intensity activities, prioritizing higher frequencies of moderate activity while allowing a slightly lower threshold for vigorous activity. Participants were classified as physically active if they reported engaging in (a) vigorous activity every day, more than once a week or once a week, or (b) engaging in moderate activity every day or more than once a week; otherwise, they were considered inactive. Participants missing data for both moderate‐ and vigorous‐intensity activity were coded as missing and excluded from analyses. Similar variables classifying physical activity from 2004 to 2014 were derived for each participant.

As a sensitivity analysis, we applied a more restrictive classification of physical activity, **Benchmark PA‐2016,** previously described by Kämpfen and Maurer in HRS [26]. For this measure, participants were considered active if they reported (a) daily vigorous or moderate physical activity or both; (b) vigorous physical activity more than once a week and moderate physical activity at least 1–3 times per month; (c) vigorous physical activity once a week and moderate physical activity more than once a week; otherwise they were considered inactive. We also conducted an additional sensitivity analysis categorizing activity levels separately by intensity, **PA‐2016 intensity**. Specifically, activity level was classified as vigorous if participants reported vigorous‐intensity activity either every day or more than once a week at any moderate‐intensity activity levels; otherwise, their activity intensity level was considered moderate.

Figure S1 shows heat map illustrations of the classification of PA‐2016, PA‐2016 intensity and Benchmark PA‐2016. Details on additional covariates used in this study, defined using the RAND HRS variables [24], are provided in the Supporting Information.

### 2.4 Statistical Analysis

Using weighted linear regression models, we examined the cross‐sectional association between physical activity measured concurrently with DNAm in 2016 (PA‐2016), and each of the EAA measures in the full cohort of participants (*n* range: 3514–3850). Model 1 was adjusted for age, gender, race/ethnicity, educational attainment, total wealth, current smoker status and birth cohort. A directed acyclic diagram of the relationships with potential confounders in Model 1 is shown in Figure S2. Model 2 includes additional adjustment for BMI and mobility index, and Model 3 further adjusts for chronic disease diagnosis, all of which may act as mediators. We performed additional analyses adjusting for white blood cell counts to investigate whether the associations were confounded by variations in cell distribution. To investigate whether the associations between physical activity and EAA differed by social factors, we tested for statistical interaction by including a product term between PA‐2016 and each of the following: gender, race/ethnicity and educational attainment.

To evaluate how the timing of activity is associated with epigenetic aging, we adapted a life‐course modelling approach, SLCMA, which combines a variable selection procedure with a selective inference approach. SLCMA provides an efficient method for simultaneously testing the influence of multiple physical activity measures on EAA [19]. This approach offers a greater power to detect associations, particularly when the true underlying relationship is influenced by the timing or the cumulative amount of physical activity [27]. In the first SLCMA stage, a least angle regression (LARS) variable selection procedure was used to identify the predictor variable explaining the most variation in EAA. In the second stage, selective inference was used to estimate *p*‐values and confidence intervals (CI) for the associations between the selected variable(s) and EAA, while accounting for the variable selection process. Our SLCMA analysis included (1) time‐specific physical activity, assessed at each survey wave from 2004 to 2016 using binary classification of physical activity (active vs. inactive), to examine how the timing of physical activity relates to EAA, and (2) an accumulation measure, calculated by summing the number of survey waves in which a participant reported being physically active to provide a cumulative indicator of sustained physical activity over time, capturing its long‐term association with EAA. These measures align with life‐course approaches that distinguish between the influence of specific time periods (sensitive periods) and the cumulative effects of behaviours over time on health outcomes. For our SLCMA models, we reported findings from both a single hypothesis model, which selects the single best physical activity predictor, and a joint hypothesis model, which selects the two best physical activity predictors, allowing for the possibility for multiple measures to contribute to EAA.

We analysed two participant groups with complete follow‐up information on physical activity: (1) participants followed from 2004 to 2016 (*n* = 2334) and (2) those followed from 2010 to 2016 (*n* = 3686). This approach allowed us to account for the new cohort recruited in 2010 in the HRS and ensuring comprehensive coverage of physical activity data across both original and newly added participants. For participants with follow‐up between the 2010 and 2016 waves, we tested the association between EAA and four binary physical activity measures, as well as cumulative physical activity, or accumulation, derived as the sum of the four physical activity measures (range 0–4). Similarly, for the full cohort with follow‐up between 2004 and 2016, we tested the seven physical activity measures from the 2004 to 2016 waves, along with a cumulative physical activity measure (range 0–7).

Physical activity variables between 2004 and 2016 were correlated at 0.8 or lower (Figure S3), allowing for the variables to be tested in SLCMA [28]. Models were adjusted for age at the time of DNAm assessment, gender, race/ethnicity, educational attainment and current smoker status (2016). For each of the EAA measures, we used elbow plots to investigate the variance explained against the number of variables selected in the model, where additional variables added beyond the peak points in the plots brought diminishing returns in *R* ^2^ and complicated the interpretation of the model.

Sample characteristics were summarized using weighted means, standard deviations (SD) and percentages. Differences between groups were assessed using independent samples *t*‐tests for continuous variables and chi‐square tests for categorical variables. All statistical analyses were performed using R statistical software, in addition to the *survey*, *lars* and *selectiveInference* packages. We assessed evidence for an association using the point estimates, 95% CIs and *p*‐values < 0.05. Reported *p*‐values were two‐sided, except for those estimated for the SLCMA analysis. We also identified associations that remained significant after multiple testing correction for the epigenetic clocks at *p*‐values < 0.017.

## 3 Results

Sample characteristics by physical activity in 2016 are shown in Table 1. About 60% of the participants reported moderate‐ or vigorous‐physical activity at a frequency that met our definition (PA‐2016) and were classified as physically active. Participants who were physically active were significantly younger and more likely to be males (all *p* < 0.01). Non‐Hispanic White participants were more likely to be physically active vs. inactive (80.1% vs. 73.3%), while non‐Hispanic Black (8.4% vs. 12.8%) and Hispanic (8.2% vs. 10.3%) participants were more likely to be inactive. A higher proportion of participants with higher level of education and greater total wealth were physically active. Physically active participants were more likely to be non‐smokers and to have lower BMI, better mobility and lower prevalence of chronic conditions.

**TABLE 1:** Sample characteristics by physical activity status in the Health and Retirement Study (2016).

	Physical activity (PA‐2016)^a^		*p*
Sample characteristics	Inactive	Active	
Mean (SD) or %	*n* = 1620	*n* = 2253	
Age (years)	70.4 (10.0)	67.4 (8.5)	< 0.01
Female gender	60.1	50.4	< 0.01
Race/ethnicity			< 0.01
Non‐Hispanic White	73.3	80.1	
Non‐Hispanic Black	12.8	8.4	
Hispanic	10.3	8.2	
Other	3.6	3.3	
Educational attainment			< 0.01
Less than high school	18.0	8.8	
High school or equivalent	58.0	46.1	
Some college or higher	24.1	45.1	
Total wealth ($100 K)	3.6 (8.9)	7.3 (16.2)	< 0.01
Current smoker status	13.5	9.6	< 0.01
Body mass index (kg/m^2^)	31.5 (7.3)	29.4 (6.3)	< 0.01
Mobility index (0–5)	1.8 (1.7)	0.7 (1.1)	< 0.01
Chronic disease diagnosis	79.4	65.3	< 0.01
Birth cohort			< 0.01
Older cohort	53.0	39.0	
Cohort‐2004	21.3	26.9	
Cohort‐2010	25.7	34.1	

Abbreviation: PA, physical activity.

^a^ Physical activity status missing due to missing information on moderate‐ and vigorous‐intensity activity (*n* = 2).

### 3.1 Cross‐Sectional Analysis Results

Table 2 shows the association between each of the EAA measures and PA‐2016. In Model 1, we adjusted for age, gender, race/ethnicity, educational attainment, total wealth, current smoker status and birth cohort. In Model 1, compared to participants who were not physically active, being physically active was associated with a decrease in GrimAA, with an effect estimate equivalent to 1.3 years in physically active participants (*b* = −1.26, 95% CI: −1.59, −0.93). In Model 2, adjusting for BMI and mobility index attenuated the effect estimate to −0.78 years (95% CI: −1.11, −0.44). In Model 3, which further adjusted for chronic disease diagnosis, being physically active remained significantly associated with a change in GrimAA equivalent to −0.76 years (95% CI: −1.09, −0.42). For PhenoAA, we observed similar associations where being physically active was associated with slower EAA equivalent to 1.70 years (95% CI: −2.26, −1.15) in Model 1, −1.29 years (95% CI: −1.87, −0.71) in Model 2 and −1.26 years (95% CI: −1.84, −0.68) in the fully adjusted Model 3. PACE was also associated with a decrease in the rate of biological aging equivalent to 0.05 years for every year of chronological age (95% CI: −0.06, −0.04) in Model 1 and −0.03 (95% CI: −0.04, −0.02) in Models 2 and 3. All associations remained significant after accounting for multiple testing.

**TABLE 2:** Cross‐sectional regression results for epigenetic age acceleration measures in the Health and Retirement Study (2016).

	GrimAA			PhenoAA			PACE		
	Effect estimate (95% CI)			Effect estimate (95% CI)			Effect estimate (95% CI)		
	Model 1	Model 2	Model 3	Model 1	Model 2	Model 3	Model 1	Model 2	Model 3
Physically active	−1.26 ***	−0.78 ***	−0.76 ***	−1.70 ***	−1.29 ***	−1.26 ***	−0.05 ***	−0.03 ***	−0.03 ***
(vs. inactive, PA‐2016)	(−1.59, −0.93)	(−1.11, −0.44)	(−1.09, −0.42)	(−2.26, −1.15)	(−1.87, −0.71)	(−1.84, −0.68)	(−0.06, −0.04)	(−0.04, −0.02)	(−0.04, −0.02)
Age (years)	0.01 (−0.02, 0.04)	0.01 (−0.02, 0.04)	0.01 (−0.02, 0.04)	0.02 (−0.04, 0.08)	0.02 (−0.04, 0.09)	0.02 (−0.04, 0.08)	0.001 * (0.0002, 0.002)	0.002 *** (0.001, 0.003)	0.002 *** (0.001, 0.003)
Female gender	−3.33 *** (−3.63, −3.03)	−3.42 *** (−3.72, −3.12)	−3.37 *** (−3.67, −3.07)	−1.27 *** (−1.80, −0.73)	−1.33 *** (−1.87, −0.80)	−1.28 *** (−1.81, −0.74)	−0.04 *** (−0.05, −0.03)	−0.04 *** (−0.05, −0.03)	−0.04 *** (−0.05, −0.03)
Race/ethnicity									
Non‐Hispanic White	Ref.	Ref.	Ref.	Ref.	Ref.	Ref.	Ref.	Ref.	Ref.
Non‐Hispanic Black	1.20 *** (0.72, 1.68)	1.12 *** (0.65, 1.59)	1.03 *** (0.57, 1.50)	0.45 (−0.46, 1.36)	0.38 (−0.53, 1.29)	0.29 (−0.63, 1.20)	0.07 *** (0.06, 0.09)	0.07 *** (0.05, 0.09)	0.07 *** (0.05, 0.08)
Hispanic	−0.49 (−1.00, 0.01)	−0.55 * (−1.05, −0.05)	−0.59 * (−1.09, −0.09)	−0.39 (−1.29, 0.51)	−0.44 (−1.33, 0.46)	−0.48 (−1.38, 0.41)	0.04 *** (0.02, 0.06)	0.04 *** (0.02, 0.05)	0.03 *** (0.02, 0.05)
Other	0.18 (−0.60, 0.95)	0.11 (−0.66, 0.89)	0.06 (−0.71, 0.82)	0.29 (−1.15, 1.73)	0.26 (−1.15, 1.66)	0.20 (−1.21, 1.60)	0.02 (−0.01, 0.05)	0.02 (−0.01, 0.05)	0.02 (−0.01, 0.05)
Educational attainment									
Less than high school	Ref.	Ref.	Ref.	Ref.	Ref.	Ref.	Ref.	Ref.	Ref.
High school or equivalent	−0.45 (−0.96, 0.05)	−0.37 (−0.87, 0.13)	−0.36 (−0.86, 0.15)	−0.05 (−0.88, 0.78)	−0.001 (−0.83, 0.83)	0.02 (−0.81, 0.84)	−0.02 ** (−0.04, −0.01)	−0.02 ** (−0.04, −0.01)	−0.02 ** (−0.04, −0.01)
Some college or higher	−1.57 *** (−2.14, −1.00)	−1.38 *** (−1.94, −0.82)	−1.34 *** (−1.90, −0.78)	−0.34 (−1.28, 0.60)	−0.20 (−1.14, 0.75)	−0.17 (−1.11, 0.77)	−0.05 *** (−0.07, −0.04)	−0.05 *** (−0.06, −0.03)	−0.04 *** (−0.06, −0.03)
Total wealth ($100 K)	−0.02 (−0.04, 0.00)	−0.01 (−0.03, 0.01)	−0.01 (−0.03, 0.01)	−0.02 ** (−0.03, −0.01)	−0.02 * (−0.03, −0.002)	−0.02 * (−0.03, −0.002)	−0.001 *** (−0.001, −0.0004)	−0.001 ** (−0.001, −0.0002)	−0.001 ** (−0.001, −0.0002)
Current smoker status	6.17 ***	6.22 ***	6.22 ***	0.70	0.84	0.82	0.08 ***	0.09 ***	0.09 ***
(vs. non‐smoker)	(5.65, 6.69)	(5.69, 6.75)	(5.69, 6.75)	(−0.13, 1.53)	(−0.01, 1.69)	(−0.03, 1.67)	(0.06, 0.09)	(0.08, 0.11)	(0.08, 0.11)
Birth Cohort									
Older cohort	Ref.	Ref.	Ref.	Ref.	Ref.	Ref.	Ref.	Ref.	Ref.
Cohort‐2004	−0.50 (−1.01, 0.02)	−0.45 (−0.97, 0.06)	−0.44 (−0.95, 0.07)	0.47 (−0.48, 1.41)	0.53 (−0.42, 1.48)	0.55 (−0.40, 1.50)	−0.02 * (−0.04, −0.00)	−0.01 (−0.03, 0.00)	−0.01 (−0.03, 0.00)
Cohort‐2010	−0.34 (−0.98, 0.30)	−0.29 (−0.93, 0.34)	−0.22 (−0.85, 0.42)	0.64 (−0.56, 1.85)	0.74 (−0.47, 1.95)	0.83 (−0.39, 2.04)	−0.02 (−0.05, 0.00)	−0.01 (−0.03, 0.01)	−0.01 (−0.03, 0.01)
BMI (kg/m^2^)		0.04 ** (0.01, 0.06)	0.03 * (0.01, 0.06)		0.06 ** (0.01, 0.10)	0.05 * (0.01, 0.09)		0.01 *** (0.00, 0.01)	0.005 *** (0.004, 0.014)
Mobility index (0–5)		0.40 *** (0.28, 0.53)	0.37 *** (0.25, 0.50)		0.29 ** (0.08, 0.50)	0.26 * (0.05, 0.47)		0.01 *** (0.01, 0.02)	0.01 *** (0.01, 0.01)
Chronic disease diagnosis (vs. no chronic disease)			0.71 *** (0.36, 1.07)			0.67 * (0.02, 1.32)			0.02 *** (0.01, 0.04)

*Note:* Covariates measured in 2016, except for BMI, which was calculated using measured weight in 2014 and 2016. Effect estimate represents the change in age acceleration (for GrimAA and PhenoAA) or the rate of biological aging per 1 year of chronological age (for PACE) associated with being physically active vs. not being physically active. All results for main exposure (PA‐2016) meet the Bonferroni‐adjusted significance threshold (*p* < 0.017).

Abbreviations: BMI, body mass index; CI, confidence interval; PA, physical activity.

*** *p*‐value < 0.001.

** *p*‐value < 0.01.

* *p*‐value < 0.05.

**FIGURE 1:**
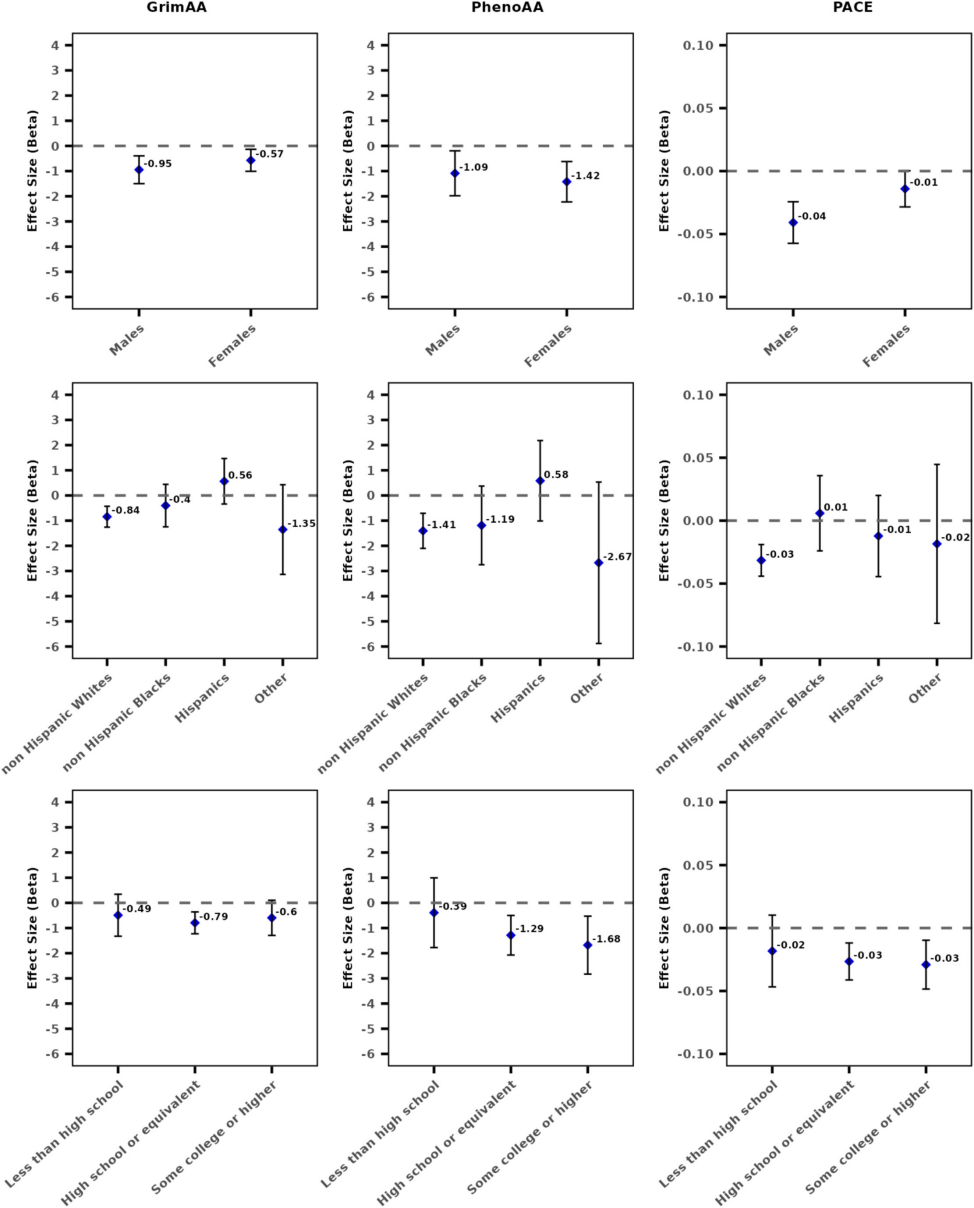
Cross‐sectional associations between physical activity (PA‐2016) and epigenetic age acceleration measures in Model 3 stratified by (a) gender, (b) race/ethnicity and (c) educational attainment in the Health and Retirement Study (2016).

Table 3 shows the significant effect modification results of the association between PA‐2016 and EAA by social factors. Compared to females, males showed a greater magnitude of slower rate of biological aging measured using PACE with physical activity, but the association was not significant after accounting for multiple testing (*P*_interaction_ = 0.04). For race/ethnicity, physical activity was associated with increased GrimAA in Hispanic participants (*P*_interaction_ = 0.009) compared to non‐Hispanic Whites. The association between physical activity and EAA was generally consistent across educational attainment levels, though it was slightly stronger among participants with higher educational attainment for PhenoAA. Associations from models stratified by social factors, adjusted for covariates in Model 3, are presented in Figure 1.

**TABLE 3:** Significant effect modification of the association between physical activity and epigenetic age acceleration measures by social factors in the Health and Retirement Study (2016).

Epigenetic age acceleration	Interaction term	Main effect		Interaction effect	
		Effect estimate (SE)	*p*	Effect estimate (SE)	*p*
**PACE**	**Physically active (PA‐2016) × Gender**				
	Male (Ref.)	−0.04 (0.01)	4.0 × 10^−7^ ***		
	Female			0.02 (0.01)	0.04*
**GrimAA**	**Physically active (PA‐2016) × Race/ethnicity**				
	Non‐Hispanic White (Ref.)	−0.88 (0.20)	1.1 × 10^−5^ ***		
	Non‐Hispanic Black			0.17 (0.46)	0.67
	Hispanic			1.12 (0.43)	9.0 × 10^−3^ **
	Other			−0.25 (0.83)	0.76

*Note:* Model adjusted for age, race/ethnicity (for PACE model), gender (for GrimAA model), educational attainment, total wealth, current smoker status, body mass index, mobility index, chronic disease diagnosis and birth cohort. All results with ** *p* < 0.01 meet the Bonferroni‐adjusted significance threshold (*p* < 0.017).

Abbreviations: PA, physical activity; SE, standard error.

*** *p*‐value < 0.001.

** *p*‐value < 0.01.

* *p*‐value < 0.05.

Results from our sensitivity analysis using a more restrictive definition of activity (Benchmark PA‐2016) and examining moderate‐ and vigorous‐activity intensity separately (PA‐2016 intensity) are shown in Table S2. Using the Benchmark PA‐2016 definition, which classified 34% of participants as active, physical activity was associated with slower epigenetic aging across all models, with all associations remaining significant at the Bonferroni‐adjusted threshold (*p* < 0.017). Overall, the effect estimates for Benchmark PA‐2016 were smaller in magnitude compared to those for PA‐2016. In the analysis by intensity levels, both moderate (33% of participants) and vigorous (25%) physical activity were significantly associated with slower EAA, with stronger effects for vigorous physical activity compared to moderate physical activity (*p* < 0.017). Associations were statistically significant after accounting for multiple testing, except for the association between Benchmark PA‐2016 and PhenoAA (*p* = 0.04, Table S3). For all physical activity measures, associations with EAA remained unchanged or attenuated slightly after further adjustment for white blood cell counts.

### 3.2 SLCMA Results

Next, we investigated the association between physical activity (2010–2016 and 2004–2016) with EAA using SLCMA. Sample characteristics of participants with follow‐up over the two periods are shown in Tables S4 and S5. Over time, the proportion of current smokers decreased, while the prevalence chronic disease increased. BMI showed minimal change, whereas the mobility index increased, indicating greater mobility limitations. Among participants with 6‐year follow‐up (2010‐2016), the proportion classified as physically active ranged between 58.6% in 2016 and 60.7% in 2010. Among participants with a 12‐year follow‐up, 68.2% were classified as physically active in 2004, decreasing to 56% in 2016.

**FIGURE 2:**
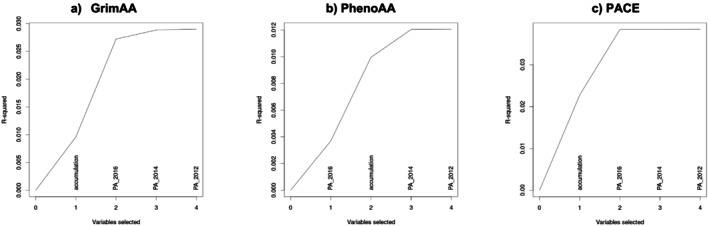
SLCMA elbow plots for the selection of physical activity variables associated with (a) GrimAA, (b) PhenoAA and (c) PACE for participants followed from 2010 to 2016 in the Health and Retirement Study. The x‐axis represents the number of variables selected, and the y‐axis shows the proportion of variance explained (*R* ^2^). The selection of variables indicates their relative importance in explaining variance in epigenetic age acceleration measures. PA, physical activity.

Tables 4 and 5 present the associations between physical activity measures and EAA in participants followed from 2010 to 2016 and 2004 to 2016, respectively. Across all three EAA measures, models consistently selected either accumulation or concurrent physical activity (PA‐2016), suggesting that sustained physical activity over multiple time points and recent physical activity contribute to explaining variations in EAA. In the 2010–2016 sample, the accumulation measure was associated with slower GrimAA, with one‐unit increase in the accumulation measure, reflecting being physically active at an additional time point, associated with *b* = −0.44 (95% CI: −0.53, −0.34) in the single hypothesis model and *b* = −0.29 (95% CI: −0.43, −0.16) in the joint hypothesis model. PA‐2016, the second best predictor, was also associated with slower GrimAA in physically active vs. inactive participants (*b* = −0.60, 95% CI: −0.98, −0.17). A similar pattern was also observed in the 2004–2016 sample. For PhenoAA, PA‐2016 was the first selected predictor, with effect estimates of *b* = −1.46 (95% CI: −1.92, −0.68) in the single hypothesis model and *b* = −0.96 (95% CI: −1.96, −0.12) in the joint hypothesis model. The accumulation measure was not significantly associated with PhenoAA in the 2010–2016 sample but showed a marginally significant association in the 2004–2016 sample (*b* = −0.24, 95% CI: −0.48, 0.02, *p* = 0.03). For PACE, accumulation was consistently associated with a slower rate of epigenetic aging across both time periods. In the 2010–2016 sample, the effect estimate for accumulation was −0.02 (95% CI: −0.02, −0.02) in the single hypothesis model and −0.01 (95% CI: −0.02, −0.01) in the joint model. PA‐2016 was the second best predictor of PACE, associated with a − 0.02 (95% CI: −0.03, −0.01) decrease in the rate of aging. In the 2004–2016 sample, accumulation remained the strongest predictor, while PA‐2016 was only marginally significant (*p* = 0.03). In the joint hypothesis models, accumulation and PA‐2016 explained 2.7% of variance (*R* ^2^) in GrimAA, 1.0% in PhenoAA and 3.8% in PACE (2010–2016) and 2.9%, 1.2% and 3.5% in the 2004–2016 sample, respectively. Figure 2 presents elbow plots visualizing the changes in the EAA variance explained with the addition of each physical activity measure to the model for the 2010–2016 sample.

**TABLE 4:** SLCMA associations between physical activity and epigenetic age acceleration measures in participants followed from 2010 to 2016 in the Health and Retirement Study (*n* = 3686).

Epigenetic age acceleration	# Of measures selected	PA measure	Effect estimate (95% CI)	*p*	*R* ^2^
GrimAA	1	Accumulation	−0.44 (−0.53, −0.34)	< 2.2 × 10^−16^ ***	1.0%
	2	Accumulation	−0.29 (−0.43, −0.16)	1.8 × 10^−5^ ***	2.7%
		PA‐2016	−0.60 (−0.98, −0.17)	5 × 10^−3^ **	
PhenoAA	1	PA‐2016	−1.46 (−1.92, −0.68)	8.4 × 10^−4^ ***	0.4%
	2	PA‐2016	−0.96 (−1.96, −0.12)	0.014 **	1.0%
		Accumulation	−0.23 (−0.48, 0.20)	0.13	
PACE	1	Accumulation	−0.02 (−0.02, −0.02)	< 2.2 × 10^−16^ ***	2.3%
	2	Accumulation	−0.01 (−0.02, −0.01)	3.0 × 10^−9^ ***	3.8%
		PA‐2016	−0.02 (−0.03, −0.01)	5 × 10^−3^ **	

*Note:* Models adjusted for age, gender, race/ethnicity, educational attainment and smoking status (2016). PA‐2016: Binary variable for physical activity status in 2016 (physically active vs. inactive). Accumulation: A cumulative measure of physical activity over multiple time points (range: 0–4), where each unit increase reflects being physically active at one additional time point. Effect estimate represents the change in age acceleration (for GrimAA and PhenoAA) or the rate of biological aging per 1 year of chronological age (for PACE) associated with being physically active vs. inactive in 2016, or one‐unit increase in physical activity accumulation. All results with ** *p* < 0.01 meet the Bonferroni‐adjusted significance threshold (*p* < 0.017).

Abbreviations: CI, confidence interval; PA, physical activity.

*** *p*‐value < 0.001.

** *p*‐value < 0.01.

* *p*‐value < 0.05.

**TABLE 5:** SLCMA associations between physical activity and epigenetic age acceleration measures in participants followed from 2004 to 2016 in the Health and Retirement Study (*n* = 2334).

Epigenetic age acceleration	# Of measures selected	PA measure	Effect estimate (95% CI)	*p*	*R* ^2^
GrimAA	1	Accumulation	−0.32 (−0.39, −0.21)	< 2.2 × 10^−16^ ***	1.0%
	2	Accumulation	−0.21 (−0.32, −0.11)	0.01 **	2.9%
		PA‐2016	−0.70 (−1.13, −0.13)	1.0 × 10^−4^ ***	
PhenoAA	1	Accumulation	−0.38 (−0.50, 0.17)	0.05	0.2%
	2	Accumulation	−0.24 (−0.48, 0.02)	0.03 *	1.2%
		PA‐2016	−0.92 (−1.79, 0.56)	0.10	
PACE	1	Accumulation	−0.01 (−0.02, −0.01)	< 2.2 × 10^−16^ ***	2.0%
	2	Accumulation	−0.01 (−0.01,‐0.01)	1.1 × 10^−6^ ***	3.5%
		PA‐2016	−0.02 (−0.04, 0.001)	0.03 *	

*Note:* Models adjusted for age, gender, race/ethnicity, educational attainment and smoking status (2016). PA‐2016: Binary variable for physical activity status in 2016 (physically active vs. inactive). Accumulation: A cumulative measure of physical activity over multiple time points (range: 0–7), where each unit increase reflects being physically active at one additional time point. Effect estimate represents the change in age acceleration (for GrimAA and PhenoAA) or the rate of biological aging per 1 year of chronological age (for PACE) associated with being physically active vs. inactive in 2016, or one‐unit increase physical activity accumulation. All results with ** *p* < 0.01 meet the Bonferroni‐adjusted significance threshold (*p* < 0.017).

Abbreviations: CI, confidence interval; PA, physical activity.

*** *p*‐value < 0.001.

** *p*‐value < 0.01.

* *p*‐value < 0.05.

## 4 Discussion

Our findings provide evidence of an association between being physically active and decreased biological aging, as measured by EAA, in a large cohort of US older adults, after adjusting for covariates including smoking, BMI, mobility limitations and chronic disease diagnoses. Using a life‐course modelling approach with longitudinally measured physical activity, we identified physical activity accumulation and concurrent physical activity as the strongest predictors of EAA. This highlights the relevance of both long‐term and recent physical activity for slowing epigenetic aging. To our knowledge, our study is one of the largest to examine this association in a diverse, US representative cohort and is the first to evaluate the temporal association between physical activity and EAA over a long follow‐up period. Our results highlight how physical activity can contribute to healthy aging through epigenetic mechanisms. This supports recommendations for physical activity to mitigate age‐related disease burden and improve the quality of life for older adults.

Prior studies examining leukocyte DNAm changes in response to physical activity indicate that inflammatory and cytokine expression pathways are key mechanisms implicated in physical activity adaptation [8, 29]. Epigenetic clocks, including those used in our study, have also been linked to inflammation [30], suggesting that physical activity may exert beneficial effects on epigenetic aging by modulating inflammatory pathways and reducing systemic inflammation. Studies on DNAm in adipose and skeletal muscle tissue suggest that physical activity influences pathways related to energy metabolism, mitochondrial function, insulin sensitivity and oxidative stress resistance, with noted differences between acute and long‐term activity [31, 32]. Further research is needed to evaluate whether the association between physical activity and epigenetic aging extends beyond inflammatory pathways, especially in relation to exercise intensity and duration.

Our findings are in line with prior studies that investigated the cross‐sectional association between epigenetic clocks and both measured [7, 8] and self‐reported physical activity [9]. In the Framingham Heart Study, each additional 5 min per day of moderate‐to‐vigorous physical activity, was associated with 19–79 days lower GrimAA, which remained significant after adjusting for BMI [7]. In participants from the Rhineland study, higher energy expenditure, step counts and time spent in moderate‐to‐vigorous intensity activities were all associated with decreased PhenoAge and GrimAge, with moderate‐to‐vigorous intensity physical activity having the strongest effect on epigenetic aging [8]. Our observed associations between physical activity and epigenetic aging slightly attenuated but remained significant after adjusting for covariates that may act as mediators, such as BMI, mobility limitations and chronic disease diagnoses, indicating alternative pathways may be at play in mediating these associations. The associations were largely unchanged after adjusting for white blood cell counts, indicating they were only minimally confounded by variations in these counts. We also observed similar associations, with smaller effect sizes, when we used a more restrictive classification of physical activity that may exclude individuals benefiting from lower frequency or intensity. This is also supported by our sensitivity analysis, which examined moderate‐ and vigorous‐physical activity separately and showed greater EAA changes with vigorous‐intensity compared to moderate‐intensity activity.

Our interaction analysis showed a consistent association between physical activity and EAA across educational attainment levels for all three clocks. For gender and race/ethnicity, we observed some evidence of significant effect modification for some of the clocks. Differences in training procedures, samples and CpGs selected could explain some the differences in clock performance [33, 34]. Physical activity was associated with slightly greater benefits in biological aging, measured using PACE, in males compared to females. Prior studies have found that females are likely to derive greater cardiovascular disease and longevity benefit from exercise, especially at lower exercise intensity [35, 36], while males may see larger improvements in specific aspects of physical performance, including muscular strength and cardiorespiratory fitness [37]. The stronger association of physical activity with PACE among males may reflect sex‐based differences in physiological adaptations to exercise, particularly in cardiorespiratory fitness. Since PACE is the only clock to incorporate lung function measures in its training procedure, these differences could partly explain our findings. We also observed increased GrimAA with physical activity in Hispanic participants compared to non‐Hispanic White and Black participants, though these findings should be interpreted cautiously due to the small Hispanic sample (*n* = 530). One potential explanation of our findings is that Hispanics in the United States are more likely to report work‐related physical activity, including occupational and transportation‐related activities, which prior studies suggest may have negative health effects compared to leisure‐time activity [38, 39].

A key strength of our study is the use of longitudinal follow‐up data to assess the relationship between being physically active, defined as engaging in moderate‐ and vigorous‐intensity activity, and EAA. Our findings highlight that both sustained and recent physical activity were significant independent predictors of slower epigenetic aging, explaining proportions of variance similar to those attributed to socioeconomic status and inflammatory markers [40, 41]. Most prior research has focused on the health benefits of structured, exercise‐based activity of relatively long duration. Emerging evidence suggests that repeated short bouts of acute‐intensity activity can trigger distinct molecular responses, leading to skeletal muscle adaptations and long‐term physiological benefits [42]. Similarly, short intermittent bouts of nonexercise physical activity of moderate‐to‐vigorous intensity in individuals followed for an average of 8 years have been linked to lower mortality and improved cardiovascular health among adults who do not engage in regular exercise [43]. These short, intermittent bouts of moderate‐to‐vigorous activity may be more feasible and accessible than structured exercise, with potential implications for physical activity recommendations and public health. While our measures do not capture the duration of the reported physical activity, our findings suggest that both sustained and concurrent activity at this level of intensity are significant predictors of epigenetic aging. Additionally, our observation that vigorous‐intensity activity was more strongly associated with slower epigenetic aging than moderate‐intensity activity aligns with these findings. Further exploration of how activity intensity, duration and patterns may shape epigenetic aging remains an important direction for future research.

Our work has limitations. Self‐reported physical activity is subject to recall bias, potentially leading to misclassification. Self‐reported physical activity may have low to moderate correlation with measured activity levels [44], yet our findings align with prior studies using accelerometer‐measured activity. Further research is needed to validate these associations using objective measures of activity. Although our analysis adjusts for key confounders such as age, gender, race and smoking, we cannot rule out residual confounding by other factors, including diet, psychological stress and genetic and lifestyle factors, which may influence both physical activity and epigenetic aging. Lastly, the SLCMA approach used to investigate the longitudinal association between physical activity and EAA does not allow for adjustment of time‐varying confounders or incorporation of complex survey design, and further research remains to address these limitations.

In conclusion, our study contributes to the growing body of evidence linking physical activity to differences in biological aging in a large, diverse and representative cohort of older US adults with long follow‐up. Our findings suggest that regular engagement in physical activity, particularly at moderate‐to‐vigorous intensity, is a robust modifiable factor that can potentially be targeted to improve biological aging. Research in this area can inform personalized recommendations, guidelines and interventions to promote overall health in aging populations.

## Ethics Statement

All participants provided informed consent to participate in the study. The University of Michigan Institutional Review Board approved the collection and analyses of these data.

## Conflicts of Interest

The authors declare no conflicts of interest.

## Supporting information


**Table S1:** Comparison of sample characteristics between the DNAm sample and the full Health and Retirement sample (2016).
**Table S2:** Cross‐sectional associations between physical activity measures (Benchmark PA‐2016 and PA‐2016 Intensity) and epigenetic age acceleration measures in the Health and Retirement Study (2016).
**Table S3:** Cross‐sectional associations between physical activity and epigenetic age acceleration measures after further adjusting for white blood cell counts in the Health and Retirement Study in 2016.
**Table S4:** Sample characteristics of participants followed from 2010 to 2016 at each follow‐up wave in the Health and Retirement Study (*n = 3686*).
**Table S5:** Sample characteristics of participants followed from 2004 to 2016 at each follow‐up wave in the Health and Retirement Study (*n = 2334*).
**Figure S1:** Classification of physical activity based on moderate‐ and vigorous‐intensity activity across different definitions: PA‐2016, PA‐2016 Intensity and Benchmark PA‐2016.
**Figure S2:** Directed acyclic diagram for the assumed relationships between physical activity, epigenetic aging and potential confounders included in Model 1.
**Figure S3:** Correlations between physical activity measures tested in SLCMA for participants followed from (a) 2010 to 2016 and (b) 2004 to 2016 in the Health and Retirement Study.
