# Supplementary material for: Physical Activity Is Associated With Decreased Epigenetic Aging: Findings From the Health and Retirement Study
Source: J Cachexia Sarcopenia Muscle. 2025 Jun 13;16(3):e13873. doi: 10.1002/jcsm.13873 (PMC12163535; doi:10.1002/jcsm.13873)
Supplement: Supplementary file 1 — Table S1: Comparison of sample characteristics between the DNAm sample and the full Health and Retirement sample (2016). Table S2: Cross‐sectional associations between physical activity measures (Benchmark PA‐2016 and PA‐2016 Intensity) and epigenetic age acceleration measures in the Health and Retirement Study (2016). Table S3: Cross‐sectional associations between physical activity and epigenetic age acceleration measures after further adjusting for white blood cell counts in the Health and Retirement Study in 2016. Table S4: Sample characteristics of participants followed from 2010 to 2016 at each follow‐up wave in the Health and Retirement Study (n = 3686). Table S5: Sample characteristics of participants followed from 2004 to 2016 at each follow‐up wave in the Health and Retirement Study (n = 2334). Figure S1: Classification of physical activity based on moderate‐ and vigorous‐intensity activity across different definitions: PA‐2016, PA‐2016 Intensity and Benchmark PA‐2016. Figure S2: Directed acyclic diagram for the assumed relationships between physical activity, epigenetic aging and potential confounders included in Model 1. Figure S3: Correlations between physical activity measures tested in SLCMA for participants followed from (a) 2010 to 2016 and (b) 2004 to 2016 in the Health and Retirement Study. [file JCSM-16-e13873-s001.docx]

**Supplementary Online Content for “Physical activity is associated with decreased epigenetic aging: Findings from the Health and Retirement Study”**

**Supplemental Methods**

Covariates

Educational attainment was categorized as a three-level variable (less than high school, high school degree/GED, and some college or higher). Total wealth, scaled to $100,000, was calculated as the sum of all wealth components (value of primary residence, secondary residence, real estate, vehicles, business, IRA, stocks, savings, bonds, and all other saving accounts) less all debts (mortgages, other home loans, and other debts). Smoking was categorized as current smoker vs. nonsmoker. For body mass index (BMI), calculated as the ratio of weight in kg to height in m^2^, we combined BMI data from 2014 and 2016 to ensure measured weight was available for the full sample. When measured BMI was missing, self-reported BMI in 2016 was used. For mobility difficulties, participants were asked about difficulty performing the following five tasks: walking several blocks, walking one block, walking across the room, climbing several flights of stairs and climbing one flight of stairs. A mobility index was derived as an aggregate score, with a scale range of 0-5, where a higher score indicates increased mobility difficulty and a score of five indicates difficulty in all 5 tasks. Finally, we derived a dichotomous measure of chronic disease diagnoses if a participant reported a physician diagnosis of type 2 diabetes, heart disease, stroke, or hypertension at any follow-up wave. The birth cohort variable included an indicator for participants' recruitment period: the older cohort (recruited prior to 2004), cohort-2004 (added in 2004, representing Early Baby Boomers born 1948-1953), and cohort-2010 (added in 2010, representing Mid Baby Boomers born 1954-1959).

**Supplemental Tables and Figures**

**Table S1: Comparison of sample characteristics between the DNAm sample and the full Health and Retirement sample (2016)**

| Sample characteristics  Mean (SD) or N (%) | All HRS sample  *n = 17,037* | DNAm sample  *n = 3,875* | *P* value |
| --- | --- | --- | --- |
| Age (years) | 64.7 (12.1) | 70.0 (9.3) | <0.01 |
| Female gender | 10,006 (58.7) | 2,241 (57.8) | 0.31 |
| Current smoker status | 2,625 (15.5) | 428 (11.1) | <0.01 |
| Birth Cohort |  |  | <0.01 |
| Older cohort | 2,622 (15.4) | 849 (21.9) |  |
| Cohort-2004 | 3,032 (17.8) | 1,027 (26.5) |  |
| Cohort-2010 | 11,383 (66.8) | 1,999 (51.6) |  |
| Reported BMI (kg/m^2^) | 29.0 (6.4) | 28.9 (6.3) | 0.25 |
| Mobility index (0-5) | 1.2 (1.6) | 1.2 (1.5) | 0.16 |
| Chronic disease diagnosis | 11,559 (68.0) | 2,916 (75.3) | <0.01 |
| Physically active  (vs. Inactive, PA-2016) | 9,685 (56.9) | 2,253 (58.2) | 0.16 |
| BMI: body mass index | | | |

**Table S2: Cross-sectional associations between physical activity measures (Benchmark PA-2016 and PA-2016 Intensity) and epigenetic age acceleration measures in the Health and Retirement Study (2016)**

|  | GrimAA  Effect estimate (95% CI) | | | PhenoAA  Effect estimate (95% CI) | | | PACE  Effect estimate (95% CI) | | |  |
| --- | --- | --- | --- | --- | --- | --- | --- | --- | --- | --- |
|  | **Model 1** | **Model 2** | **Model 3** | **Model 1** | **Model 2** | **Model 3** | **Model 1** | **Model 2** | **Model 3** |  |
| Physically active  (vs. inactive, Benchmark PA-2016) | -1.09 *** (-1.41, -0.77) | -0.63 *** (-0.96, -0.31) | -0.60 ***  (-0.92, -0.27) | -1.22 ***  (-1.78, -0.65) | -0.78 **  (-1.37, -0.19) | -0.73 *  (-1.32, -0.136) | -0.05***  (-0.06, -0.04) | -0.03 ***  (-0.04, -0.01) | -0.02***  (-0.03, -0.01) |  |
| Physically active (PA-2016 intensity) |  |  |  |  |  |  |  |  |  |  |
| Inactive | Ref. | Ref. | Ref. | Ref. | Ref. | Ref. | Ref. | Ref. | Ref. |  |
| Moderate-intensity | -0.97 ***  (-1.32, -0.62) | -0.57 **  -0.93, -0.21) | -0.56**  (-0.92, -0.20) | -1.55 ***  (-2.16, -0.95) | -1.21 ***  (-1.84, -0.58) | -1.20 ***  (-1.83, -0.58) | -0.04 ***  (-0.06, -0.03 | -0.026***  (-0.04, -0.01) | -0.03 ***  (-0.04, -0.01) |  |
| Vigorous-intensity | -1.68 ***  (-2.09, -1.27) | -1.12 ***  (-1.53, -0.70) | -1.07 ***  (-1.49, -0.66) | -1.98 ***  (-2.70, -1.28) | -1.48 ***  (-2.22, -0.74) | -1.42 ***  (-2.15, -0.68) | -0.07 ***  (-0.08, -0.05) | -0.04 ***  (-0.05, -0.03) | -0.04 ***  (-0.05, -0.02) |  |
| CI: confidence interval; PA: physical activity; BMI: body mass index  Model 1 was adjusted for age, gender, race/ethnicity, educational attainment and total wealth, current smoker status, and birth cohort. Model 2 was further adjusted for BMI and mobility index. Model 3 was further adjusted for chronic disease diagnosis.  Covariates measured in 2016, except for BMI which was calculated using measured weight in 2014 and 2016  Effect estimate represents the change in age acceleration (for GrimAA and PhenoAA) or the rate of biological aging per 1 year of chronological age (for PACE) associated with being physically active vs. inactive  *** *P* value < 0.001; ** *P* value < 0.01; * *P* value < 0.05  All results with * *P* < 0.05 meet the Bonferroni-adjusted significance threshold (*P* < 0.017) | | | | | | | | | | |

**Table S3: Cross-sectional associations between physical activity and epigenetic age acceleration measures after further adjusting for white blood cell counts in the Health and Retirement Study in 2016**

| Measure | GrimAA  Effect estimate (95% CI) | PhenoAA  Effect estimate (95% CI) | PACE  Effect estimate (95% CI) |
| --- | --- | --- | --- |
| **Physically active**  (vs. inactive, PA-2016) | -0.72 ***  (-1.07, -0.37) | -1.16 ***  (-1.76, -0.57) | -0.03 ***  (-0.04, -0.02) |
| **Physically active**  (vs. inactive, Benchmark PA-2016) | -0.51 **  (-0.84, -0.17) | -0.64 *  (-1.25, -0.03) | -0.02 ***  (-0.03, -0.01) |
| **Physically active** (PA-2016 intensity) |  |  |  |
| Inactive | Ref. | Ref. | Ref. |
| Moderate-intensity | -0.55 **  (-0.93, -0.17) | -1.10 ***  (-1.74, -0.45) | -0.02 ***  (-0.03, -0.01) |
| Vigorous-intensity | -1.00 ***  (-1.44, -0.57) | -1.33 ***  (-2.09, -0.57) | -0.03 ***  (-0.05, -0.02) |
| PA: Physical activity  Model adjusted for age, gender, race/ethnicity, educational attainment, total wealth, current smoker status, birth cohort, BMI, mobility index, chronic disease diagnosis, and white blood cell counts (B cells, CD8+ T cells, CD8+ T cells naïve, natural killer cells, and monocytes).  Effect estimate represents the change in age acceleration (for GrimAA and PhenoAA) or the rate of biological aging per 1 year of chronological age (for PACE) associated with being physically active vs. not being physically active in 2016.  *** *P* value < 0.001; ** *P* value < 0.01; * *P* value < 0.05  All results with ** *P* < 0.01 meet the Bonferroni-adjusted significance threshold (*P* < 0.017) | | | |

**Table S4: Sample characteristics of participants followed from 2010 to 2016 at each follow-up wave in the Health and Retirement Study (*n = 3,686*)**

| Sample characteristics  Mean (SD) or N (%) | 2010 | 2012 | 2014 | 2016 |
| --- | --- | --- | --- | --- |
| Age (years) | 64.3 (9.4) | 66.0 (9.4) | 67.9 (9.4) | 70.1 (9.3) |
| Total wealth ($100K) | 5.3 (9.4) | 4.0 (8.7) | 4.0 (9.2) | 4.6 (10.5) |
| Current smoking status | 533 (14.5) | 483 (13.1) | 443 (12.0) | 407 (11.0) |
| Reported BMI (kg/m^2^) | 29.1 (6.3) | 29.0 (6.3) | 29.0 (6.3) | 28.9 (6.3) |
| Type 2 diabetes | 808 (21.9) | 860 (23.4) | 948 (25.7) | 1039 (28.2) |
| Heart disease | 755 (20.5) | 839 (22.8) | 937 (25.4) | 1021 (27.7) |
| Stroke | 198 (5.4) | 222 (6.0) | 250 (6.8) | 284 (7.7) |
| Hypertension | 2,136 (58.0) | 2,238 (60.8) | 2,316 (62.9) | 2,371 (64.4) |
| Mobility index (0-5) | 1.0 (1.3) | 1.0 (1.4) | 1.1 (1.4) | 1.2 (1.5) |
| Physically active (vs. inactive,  PA-2010 to PA-2016) | 2,238 (60.7) | 2,297 (62.3) | 2,204 (59.8) | 2,161 (58.6) |
| PA: physical activity; BMI: body mass index | | | | |

**Table S5: Sample characteristics of participants followed from 2004 to 2016 at each follow-up wave in the Health and Retirement Study (*n = 2,334*)**

| Sample characteristics  Mean (SD) or N (%) | 2004 | 2006 | 2008 | 2010 | 2012 | 2014 | 2016 | |
| --- | --- | --- | --- | --- | --- | --- | --- | --- |
| Age (years) | 62.8 (7.6) | 64.8 (7.6) | 66.8 (7.6) | 69.1 (7.7) | 70.9 (7.6) | 72.8 (7.6) | 74.9 (7.6) | |
| Total wealth ($100K) | 4.3 (7.5) | 5.5 (13.3) | 5.4 (9.7) | 4.9 (9.5) | 4.9 (9.6) | 5.4 (11.1) | 5.9 (12.3) | |
| Current smoking status | 285 (12.2) | 274 (11.7) | 259 (11.1) | 233 (10.0) | 217 (9.3) | 191 (8.2) | 181 (7.8) | |
| Reported BMI, kg/m^2^ | 28.1 (5.4) | 28.4 (5.7) | 28.6 (5.8) | 28.6 (5.9) | 28.5 (5.9) | 28.4 (5.9) | 28.2 (5.8) | |
| Type 2 diabetes | 336 (14.4) | 386 (16.5) | 463 (19.9) | 544 (23.3) | 571 (24.5) | 629 (27.0) | 663 (28.4) | |
| Heart disease | 431 (18.5) | 480 (20.6) | 526 (22.5) | 581 (24.9) | 643 (27.5) | 711 (30.5) | 776 (33.3) | |
| Stroke | 79 (3.4) | 82 (3.5) | 101 (4.3) | 132 (5.7) | 156 (6.7) | 175 (7.5) | 200 (8.6) | |
| Hypertension | 1,128 (48.4) | 1,237 (53.0) | 1,369 (58.7) | 1,458 (62.6) | 1,516 (65.0) | 1,568 (67.2) | 1,594 (68.4) | |
| Mobility index (0-5) | 0.7 (1.2) | 0.8 (1.2) | 0.8 (1.2) | 1.0 (1.4) | 1.0 (1.4) | 1.1 (1.4) | 1.3 (1.5) | |
| Physical activity (vs. inactive,  PA-2004 to PA-2016) | 1,591 (68.2) | 1,612 (69.1) | 1,567 (67.1) | 1,390 (59.6) | 1,421 (60.9) | 1,358 (58.2) | 1,307 (56.0) | |
| PA: physical activity; BMI: body mass index | | | | | | | |  |

**Figure S1: Classification of physical activity based on moderate- and vigorous- intensity activity across different definitions: PA-2016, PA-2016 Intensity, and Benchmark PA-2016**


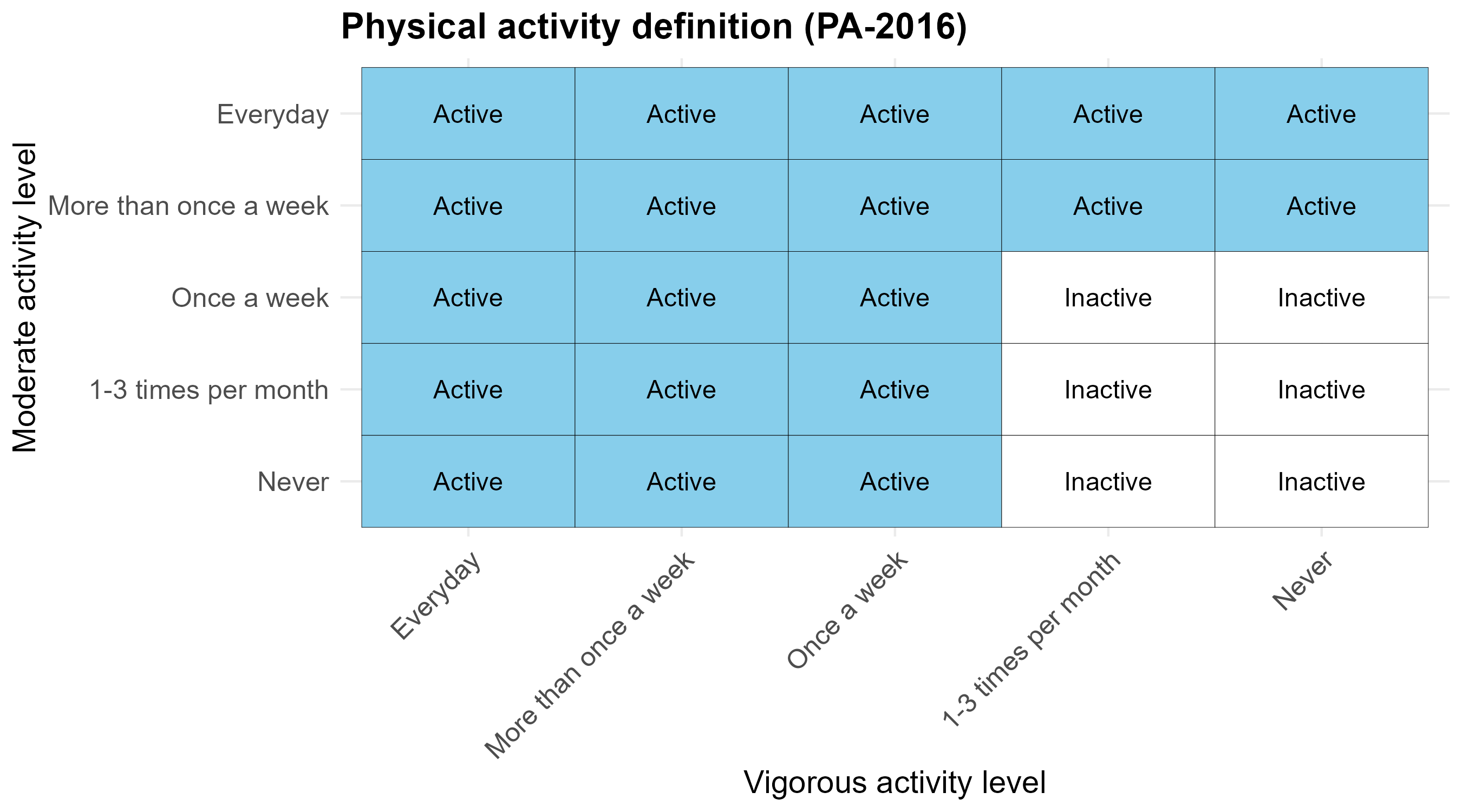

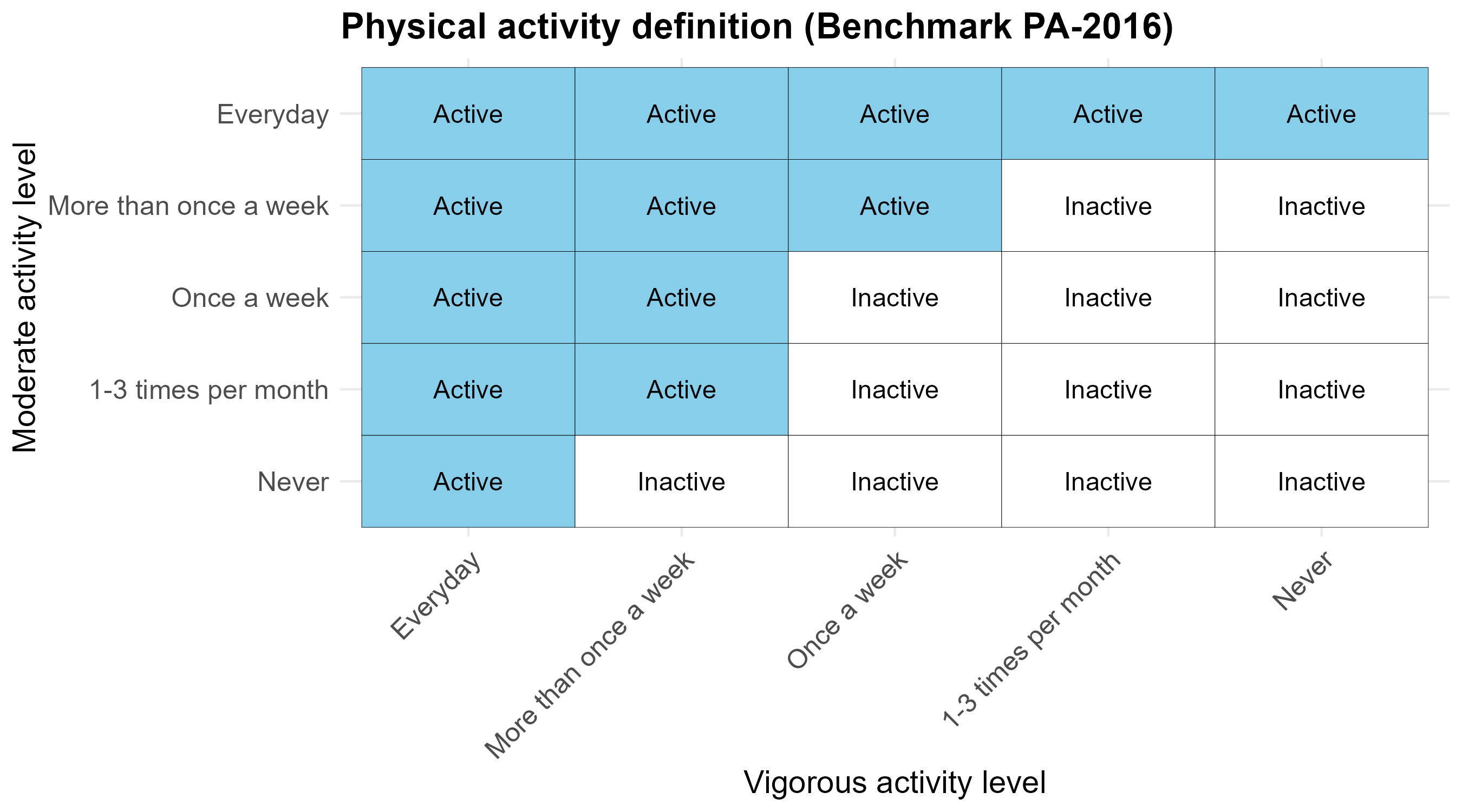

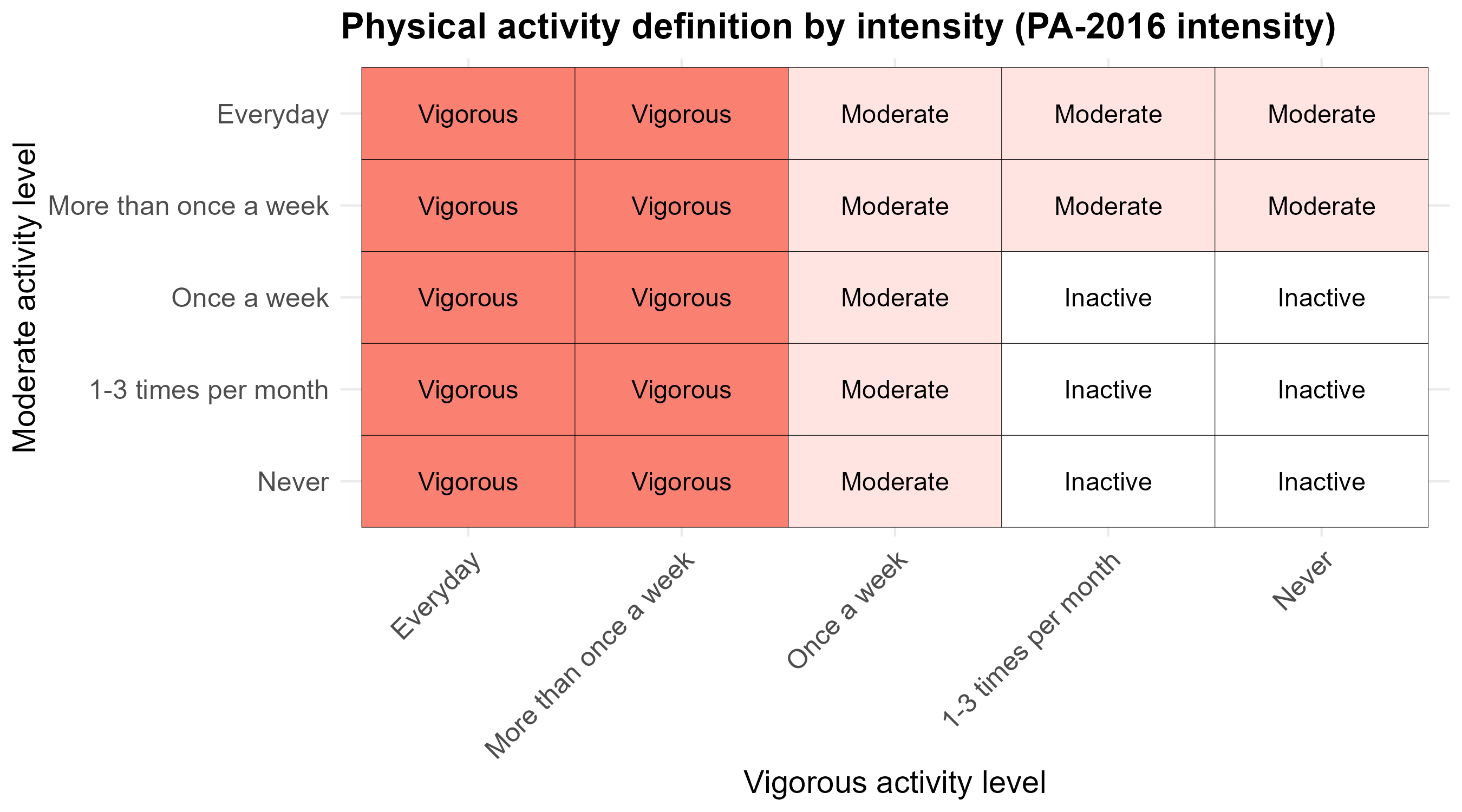


**Figure S2: Directed acyclic diagram for the assumed relationships between physical activity, epigenetic aging, and potential confounders included in Model 1**


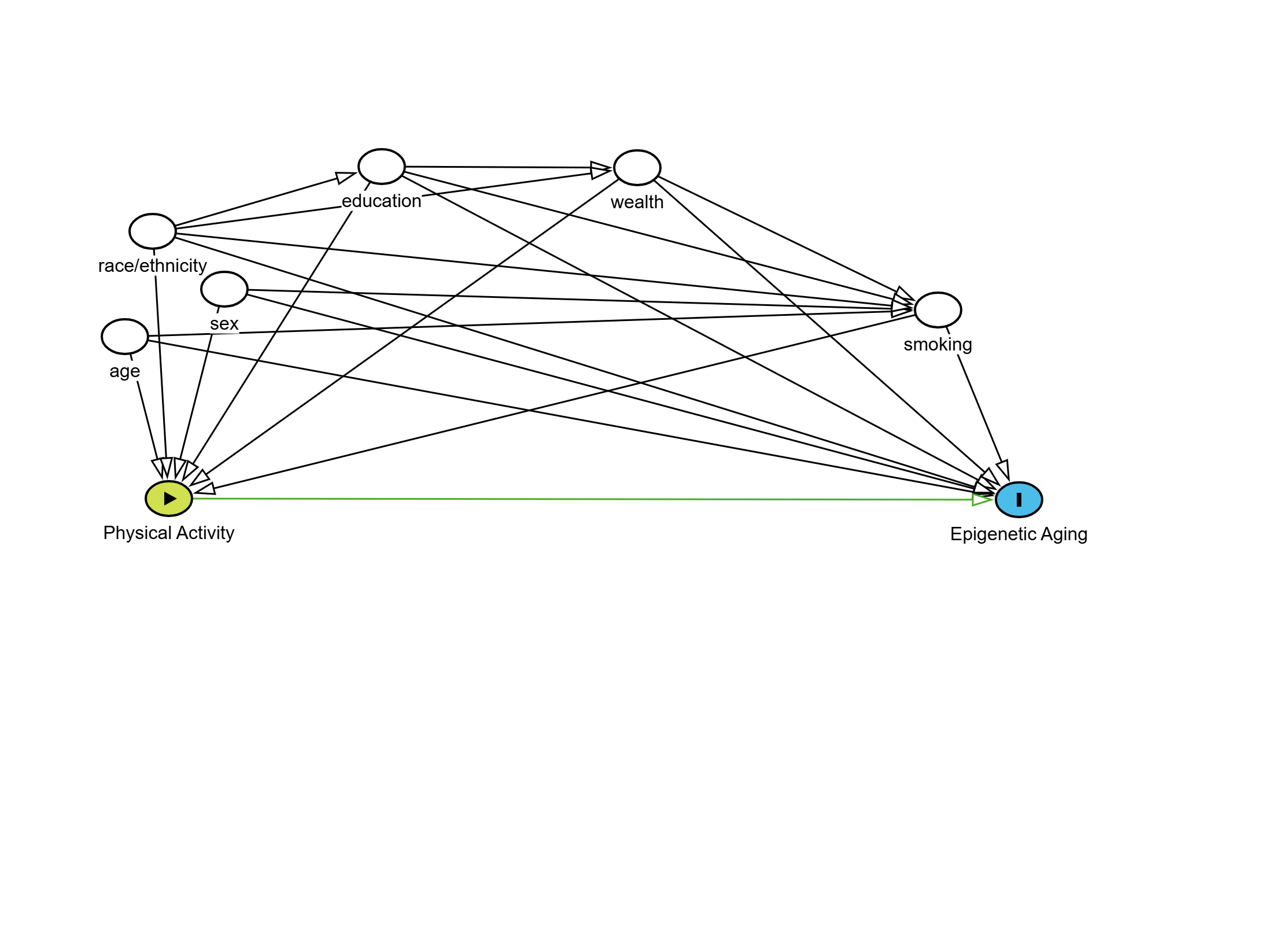


**Figure S3^[[1]](#footnote-1)^: Correlations between physical activity measures tested in SLCMA for participants followed from a) 2010 to 2016 and b) 2004 to 2016 in the Health and Retirement Study**


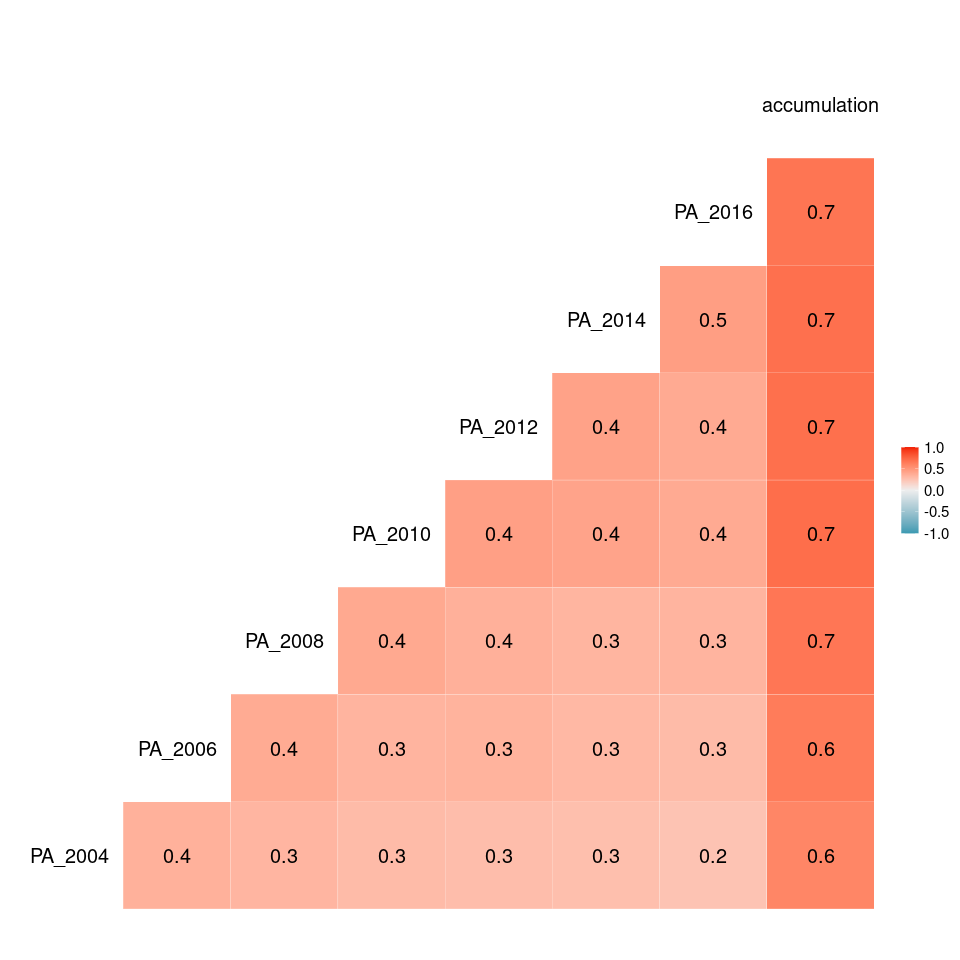

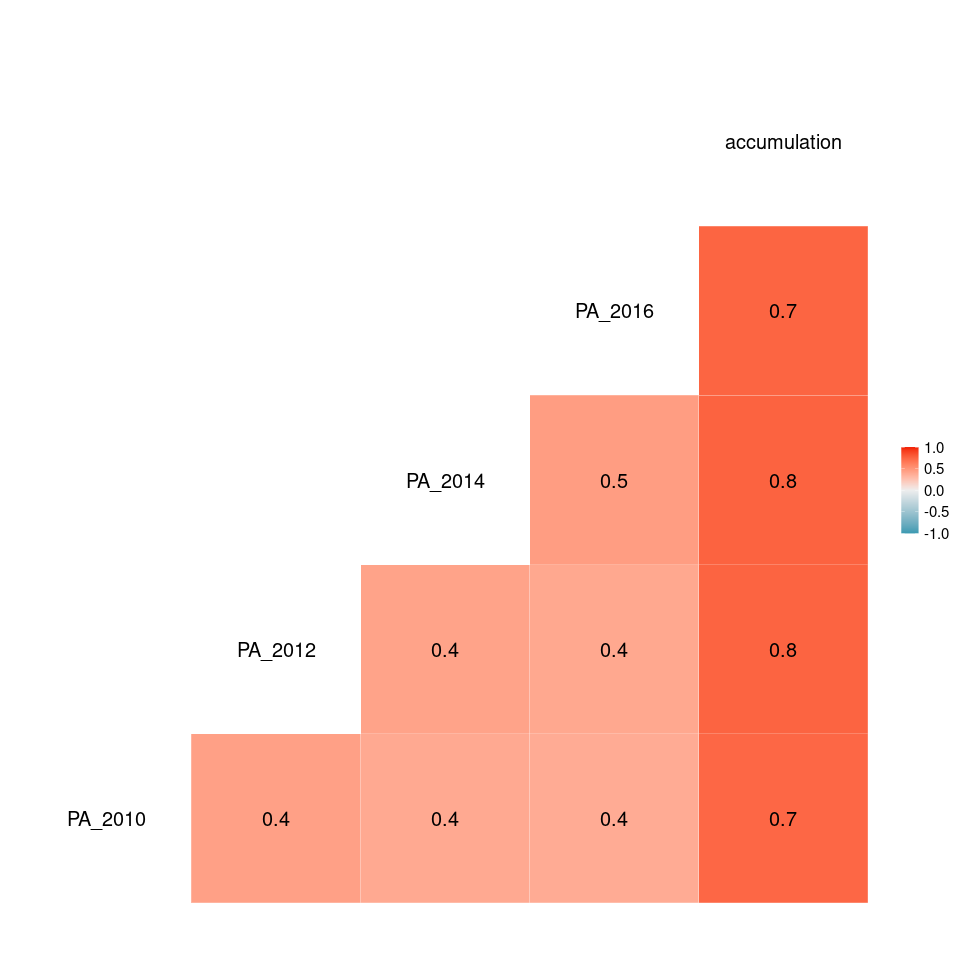


**a)**

**b)**

1. PA: physical activity [↑](#footnote-ref-1)
